# A new species of *Pilarella* (Copepoda, Calanoida, Arietellidae) from the hyperbenthic layer of Japan, with a molecular phylogenetic analysis of some representative genera of the Arietellidae

**DOI:** 10.3897/zookeys.1038.63170

**Published:** 2021-05-19

**Authors:** Sota Komeda, Kenta Adachi, Susumu Ohtsuka

**Affiliations:** 1 Takehara Station, Setouchi Field Science Center, Graduate School of Integrated Sciences for Life, Hiroshima University, 5–8–1 Minato-machi, Takehara, Hiroshima, 725–0024, Japan Hiroshima University Hiroshima Japan

**Keywords:** Arietelloidea, calanoid copepods, colonization, hyperbenthos, molecular phylogeny, Nansei Islands

## Abstract

A new species of the continental shelf hyperbenthic genus *Pilarella* is described, the first from the Indo-Pacific. This is the second species of *Pilarella* known, and the first description of a male in the genus. The new species is easily distinguished from other species of *Pilarella* (*P. longicornis*) based on: (1) short caudal rami, approximately 1.5 times longer than wide; (2) 2 setae on the mandibular endopod; (3) 6 setae on the maxillular coxal epipodite; and (4) in the female, a short left antennule reaching the posterior border of the genital double-somite. The new diagnosis of *Pilarella* differs from *Metacalanus* in the separation of ancestral segments IX–XII and XIV–XV of the antennule, and the presence of 5–6 setae on the maxillular praecoxal arthrite. *Pilarella* is also separated from *Metacalanalis* based on the absence of a seta on the third ancestral segment of the antennary exopod, the symmetry of legs 1–3, the presence of a medial basal seta on the female leg 5, and 2 lateral exopodal spines on the female leg 5. A molecular phylogenetic analysis of some representative genera of the family Arietellidae, including the present new species, recovers two arietellid clades (*Metacalanus*- and *Arietellus*-clades) as in previous studies. Dichotomous keys for the genera of Arietellidae and the species of *Pilarella* are included.

## Introduction

The calanoid family Arietellidae is widely distributed, occurring from littoral caves to the shallow water pelagic realm and deep-sea hyperbenthic layers (Ohtsuka et al. 1993, 1994). Phylogenetic analysis of the family pointed to the existence of two clades, viz. *Crassarietellus*-*Paramisophria*-*Metacalanus*-*Pilarella* (*Metacalanus*-clade) and *Campaneria*-*Scutogerulus*-*Paraugaptilus*-*Paraugaptiloides*-*Arietellus*-*Sarsarietellus* (*Arietellus*-clade) (Ohtsuka et al. 1994). Subsequently, Ohtsuka et al. (2005) and Soh et al. (2013) reconstructed the phylogenetic relationships among genera adding characters of new taxa such as *Protoparamisophria*, *Metacalanalis* and *Paraugaptiloides*. According to Soh et al. (2013), the position of *Paramisophria* is equivocal, while *Crassarietellus*-*Metacalanalis*-*Metacalanus*-*Pilarella*-*Scutogerulus*-*Protoparamisophria* and *Campaneria*-*Paraugaptilus*-*Arietellus*-*Paraugaptiloides*-*Sarsarietellus* belong to Ohtsuka et al.’s (1994)*Metacalanus*- and *Arietellus*-clades, respectively. These studies (Ohtsuka et al. 1994, 2005; Ohtsuka 2006; Soh et al. 2013) implied the different colonization routes followed by the family and suggested that pelagic taxa (some species of *Metacalanus* in *Metacalanus*-clade, and *Arietellus* and *Paraugaptilus* in *Arietellus*-clade) could have been independently derived from hyperbenthic forms in each clade.

Recently, molecular markers such as ribosomal and mitochondrial DNA sequences have been used as tools for the exploration of phylogenetic relationships among various calanoid taxa. However, only data on one arietellid species (*Paraugaptilus buchani*) is available in the DDBJ/EMBL/GenBank databases, and no molecular phylogenetic analysis has been conducted yet among arietellid genera.

This study describes a new species of *Pilarella* collected off the Amami Oshima Island, Kagoshima Prefecture, Japan. This is the second species of *Pilarella* and the first record of this genus in the Indo-Pacific. The male of *Pilarella* is described for the first time. Moreover, we explore the phylogenetic relationships among seven arietellid species based on 28S ribosomal DNA. Furthermore, new dichotomous keys to arietellid genera and species of *Pilarella* are provided.

## Materials and methods

### Sample collection and morphological observations

The arietellid copepods used in this study were collected off the Nansei Islands in southwest Japan in May 2018 and 2019 by the training and research vessel “*Toyoshio-Maru*” of Hiroshima University. Sampling dates, localities, and collection gears (cf. Ohtsuka et al. 1992) are shown in Table 1. Specimens were fixed in 99.5% ethanol.

**Table 1.** Collection data of arietellid species used in molecular phylogeny.

Species	Accession number	Sampling gear	Longitude / Latitude	Depth (m)	Date	Time
*Arietellus setosus* Giesbrecht, 1892	LC510290	ORI net	29°50.011'N, 130°55.999'E	0–1000	May 22, 2019	1635–1912
*Arietellus simplex* Sars, 1905	LC510291	ORI net	29°50.011'N, 130°55.999'E	0–1000	May 22, 2019	1635–1912
*Metacalanus* sp.	LC516702	Conical hand net	26°23'N, 127°67'E	<10	May 25, 2019	Night
*Paramisophria* sp.	LC510294	Dredge	26°14.448'N, 127°31.532'E	53	May 25, 2019	0909–1020
*Paraugaptilus buchani* Wolfenden, 1904	LC510293	ORI net	30°48.106'N, 131°32.072'E	0–1000	May 15, 2018	0941–1205
*Pilarella compacta* sp. nov.	LC510295	Sledge net	28°14.023'N, 129°39.559'E	291–294	May 18, 2018	0816–0826
*Sarsarietellus orientalis* Soh, Moon, Ohtsuka, Pae & Jeong, 2013	LC510292	Beam trawl nets	28°22.422'N, 129°15.144'E	315–316	May 23, 2019	1155–1255

Specimens of the new species of *Pilarella* were dissected under a stereomicroscope (SZX7, Olympus), and their appendages and urosome cleared in lactophenol. Illustrations were drawn under a biological microscope (BX53, Olympus) with a drawing tube.

Type specimens (NMST-Cr29010–29012) were deposited at the National Museum of Nature and Science (Tsukuba, Ibaraki Prefecture, Japan). The morphological terminology mainly follows Huys and Boxshall (1991); alternative interpretations of the homology of the maxilla and maxilliped follow Ferrari and Ivanenko (2001, 2008).

### DNA extraction, PCR, and sequencing

Total DNA of samples, except of *Metacalanus* sp., was extracted from the whole body using DNeasy Blood & Tissue kits (Qiagen, Venlo, Netherlands). Total DNA extraction from a small sample of *Metacalanus* sp. was performed using the whole body according to the method described by Suyama (2011). DNA was quantified using a NanoDrop 2000 instrument (Thermo-Fisher, Waltham, MA, USA) and then adjusted to 1 ng μL^– 1^ with sterilized water for PCR amplification. The 28S nuclear ribosomal DNA region (*28S*) was amplified using a T100 Thermal Cycler (Bio-Rad, Hercules, CA, USA) with Taq PCR Master Mix Kit (Qiagen) and primers; 28S-F1a (5ʹ-GCG GAG GAA AAG AAA CTA AC-3ʹ) and 28S-R1a (5ʹ-GCA TAG TTT CAC CAT CTT TCG GG-3ʹ) (Blanco-Bercial et al. 2011). Thermocycling conditions for *28S* were 94 °C for 7 min; 35–40 cycles at 94 °C for 45 s, 50 °C for 1 min, and 72 °C for 1 min; and a final extension at 72 °C for 7 min. Amplification results were verified using 2% (w/v) agarose electrophoresis. Excess primers and dNTPs were removed with ExoSAP-IT (Thermo-Fisher, Scientific, Waltham, MA, USA), and the sequencing was performed commercially (Macrogen Japan, Kyoto, Japan).

Seven sequences for *28S* (Accession numbers: LC510290–LC510295, LC516702) obtained in this study were deposited in the DNA Data Bank of Japan (DDBJ) and GenBank. *Paraheterorhabdus compactus* (Sars, 1900) (Accession number: HM997026) was used as the outgroup taxon. Sequence alignment was performed using CLUSTAL W (Thompson et al. 1994) in MEGA 7.026 (Tamura et al. 2011), and final alignments of 649 bp were used for the phylogenetic analysis. Phylogenetic relationships among the partial *28S* sequences were inferred using Maximum Likelihood (ML) and Bayesian Inference (BI) analyses. ML analysis was performed using software RAxML 8 (Stamatakis 2006, Stamatakis et al. 2008) under the GTR+Γ model, and the data set was run with 10,000 bootstrap replicates. BI analysis was performed using the software MrBayes 3.2.7 (Ronquist et al. 2012) under the GTR+Γ model. Two parallel analyses of Metropolis-Coupled Markov Chain Monte Carlo (MC_3_) were conducted for 1,000,000 generations, and topologies were sampled every 100 generations. The convergence of MCMC was checked with the value of the average standard deviation of split frequencies (ASDSF) in MrBayes and trace plots in the software Tracer 1.7.1 (Rambaut et al. 2018). The first 2500 trees (25% of all trees) were discarded as burn-in, and the consensus was estimated by summarizing the remainder 7500 trees. The phylogenetic trees of ML and BI analyses were visualized with Figtree 1.4.4 (Rambaut 2009).

## Results

### Taxonomy

#### Order Calanoida Sars, 1901

**Family Arietellidae Sars, 1901**

##### Genus Pilarella

Taxon classificationAnimaliaCalanoidaArietellidae

Alvarez, 1985

3C51F783-6E0E-56C4-B823-E4ABEE3A0D85

###### Diagnosis.

**Female.** Cephalosome separated from first pediger. Fourth and fifth pedigers completely fused. Rostrum produced ventrally, with pair of frontal filaments disposed distally. Genital double-somite symmetrical with paired seminal receptacles and gonopores located ventrolaterally. Antennules asymmetrical, 21-segmented, ancestral segments I-IV and XXIV-XXVIII fused. Left antennule exceeding fifth pediger, approximately 1.5 times longer than right counterpart. Antenna with unarmed coxa; exopod 5-segmented, with ancestral segments II-IV, V-VII and IX-X fused, setal formula as 0, 0-0-1, 1-1-1, 1, 0-2. Mandible with row of setules on dorsal margin of gnathobase; endopod rudimentary, unsegmented, with 2 setae. Maxillule with 5–6 spines on praecoxal arthrite; coxal endite with 1 seta; coxal epipodite with 5–6 setae; proximal and distal basal endites without seta; endopod unsegmented with 2 setae; exopod with 3 setae. Maxilla with 2 setae on all praecoxal and coxal endites; basis having 1 heavily-chitinized spine [Maxilla with 2 setae on all praecoxal, coxal and basal endites; first endopodal segment having 1 heavily-chitinized spine (the homology by Ferrari and Ivanenko 2001, 2008)]. Maxilliped syncoxal endites with 0, 1, 0, and 2 setae, respectively; basis with 2 setae; first to sixth endopodal segments with 1, 4, 3, 2, 2 and 4 setae, respectively [Maxilliped praecoxal endites with 0, 1 and 0 setae, respectively; coxal endite with 2 setae; basis with 2 setae midway and 1 seta distally; first to fifth endopodal segments with 4, 3, 2, 2 and 4 setae, respectively (the homology by Ferrari and Ivanenko 2001, 2008)]. Legs 1–4 symmetrical; Seta and spine formula shown in Table 2, but basal lateral seta of leg 4 reduced in *P. longicornis*. Leg 5 uniramous, 3-segmented; basis with long medial seta and short lateral seta; exopod unsegmented, with 1 lateral spine and 2 terminal spines.

**Table 2.** Setal formula of legs 1–4 of *Pilarella compacta* sp. nov. Roman numerals: spines, Arabic numerals: setae.

			Exopod			Endopod		
	Coxa	Basis	1	2	3	1	2	3
Leg 1	0–1	1–1	I-1;	I-1;	0, I, 5	0–1;	0–2;	1, 2, 2
Leg 2	0–1	0–0	I-1;	I-1;	III, I, 5	0–1;	0–2;	2, 2, 4
Leg 3	0–1	0–0	I-1;	I-1;	III, I, 5	0–1;	0–2;	2, 2, 4
Leg 4	0–1	1–0	I-1;	I-1;	III, I, 5	0–1;	0–2;	2, 2, 3

**Male.** Body, antenna, mandible, maxillule, maxilla, maxilliped, and legs 1–4 similar to female counterparts. Antennules asymmetrical; right antennule 21-segmented, ancestral segments I–IV fused, XXIV–XXVIII partly fused, I–VIII with long tape-like aesthetascs. Leg 5 uniramous, 5-segmented; basis with lateral seta; exopod 3-segmented, proximal 2 segments with 1 lateral spine, distal segment with 2 terminal setae.

###### Type species.

*Pilarella longicornis* Alvarez, 1985 (by monotypy). Other species: *Pilarella compacta* sp. nov., described herein.

###### Remarks.

The diagnosis of *Metacalanus* from Ohtsuka et al. (1994) differs from *Pilarella* in the fusion of ancestral segments IX–XII and XIV–XV on the antennule, and the display of 0–2 setae on the maxillular praecoxal arthrite (vs. 5–6 setae in *Pilarella*). The diagnosis of *Metacalanalis* from Ohtsuka et al. (2005) differs also from *Pilarella* in the presence of 1 seta on the third ancestral segment of the antennary exopod, the asymmetry of legs 1–3, the absence of medial basal seta on female leg 5, and the presence of 3 lateral exopodal spines on the female leg 5 (vs. 2 in *Pilarella*).

##### Pilarella compacta sp. nov.

Taxon classificationAnimaliaCalanoidaArietellidae

DD5DF73D-DA9F-5DFE-88F3-D5F017594A03

http://zoobank.org/CD13E6FD-04D7-42EE-9CDB-571B0E9DB325

###### Types.

***Holotype***: ♀ 1.97 mm preserved in vial (NMST-Cr29010). ***Allotype***: ♂ 1.69 mm, appendages mounted on glass slide, body in vial (NMST-Cr29011). ***Paratype***: ♀ 2.01 mm, appendages mounted on glass slide, body in vial (NMST-Cr29012).

###### Description.

**Figure 1.**
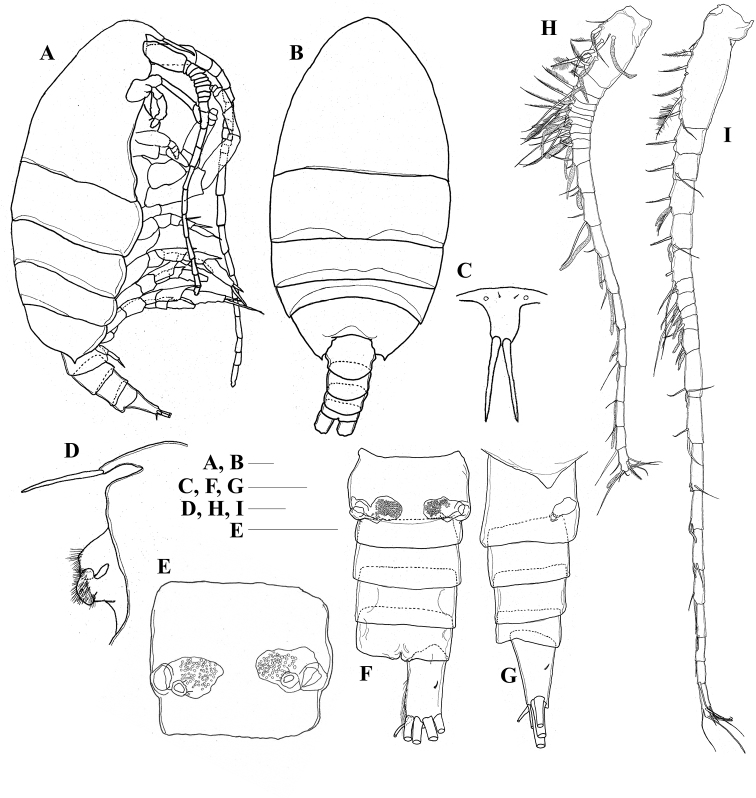
*Pilarella compacta* sp. nov., adult female **A** lateral habitus (holotype) **B** dorsal habitus (holotype) **C** rostrum, ventral view (paratype) **D** rostrum, labrum and left paragnath, lateral view (paratype) **E** genital double-somite, ventral view (paratype) **F** urosome, ventral view (paratype) **G** urosome, lateral view (paratype) **H** right antennule (paratype) **I** left antennule (paratype). Scale bars: 0.1 mm.

**Adult female. *Body*** (Fig. 1A, B) compact; cephalosome separated from first pediger; fourth and fifth pedigers completely fused; posterolateral corners of prosome with small triangular process extending posteriorly. Rostrum (Fig. 1C, D) produced ventrally, with pair of frontal filaments distally. Urosome (Fig. 1E–G) 4-segmented; genital double-somite symmetrical with pair of seminal receptacles; pair of gonopores located ventrolaterally. Caudal rami (Fig. 1F, G) symmetrical, approximately 1.5 times longer than wide; minute seta I placed ventrally, seta II reduced, setae III–VI long, short seta VII dorsally; inner margin of rami furnished with row of fine setules.

***Antennules*** (Fig. 1H, I) asymmetrical, left antennule approximately 1.5 times longer than right antennule. Right antennule (Fig. 1H) 21-segmented, reaching middle of second pediger, fusion pattern and armature of segments as follows: I-IV–9+2ae, V–1+1ae, VI–2, VII–2+1ae, VIII–2+1ae, IX–1+1ae, X–1+1ae, XI–2+1ae, XII–2+1ae, XIII–2+1ae, XIV–2+1ae, XV–2+1ae, XVI–2+1ae, XVII–2+1ae, XVIII–2+1ae, XIX–1+1ae, XX–2+1ae, XXI–1+1ae, XXII–1, XXIII–1, XXIV-XXVIII–10+1ae. Left antennule (Fig. 1I) 21-segmented, reaching posterior border of genital double-somite, fusion pattern and armature of segments as follows: I-IV–8, V–1, VI–2, VII–1+1ae, VIII–2+1ae, IX–2, X–2, XI–2+1ae, XII–2+1ae, XIII–1+1ae, XIV–2+1ae, XV–2+1ae, XVI–2, XVII–2+1ae, XVIII–2+1ae, XIX–1+1ae, XX–2, XXI–2+1ae, XXII–1, XXIII–1, XXIV-XXVIII–9.

**Figure 2.**
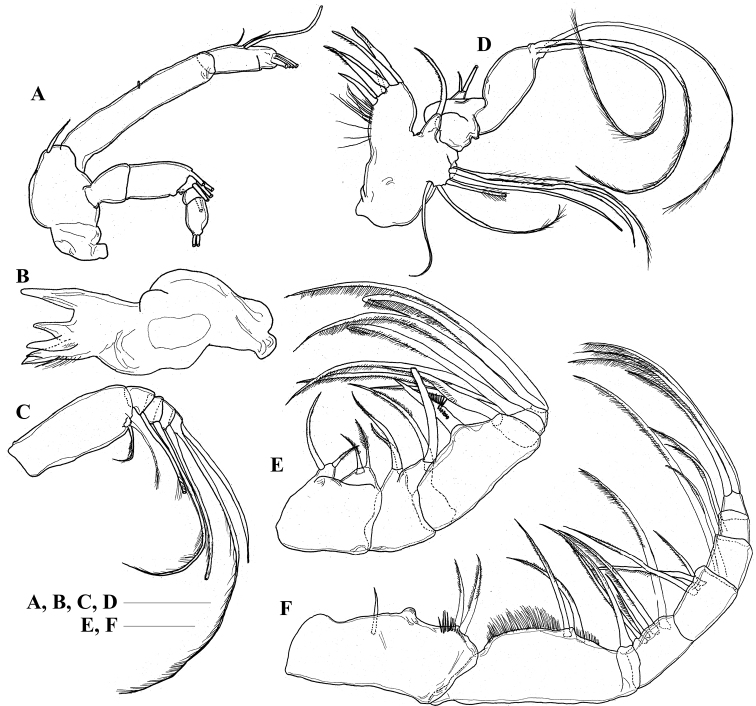
*Pilarella compacta* sp. nov., adult female, paratype **A** right antenna **B** coxa of left mandible **C** palp of left mandible **D** left maxillule **E** right maxilla **F** right maxilliped. Scale bars: 0.1 mm.

***Antenna*** (Fig. 2A) with unarmed coxa; basis with inner distal seta; exopod 5-segmented; ancestral segments II-IV, V-VII and IX-X fused, setal formula of 0, 0-0-1, 1-1-1, 1, 0-2; endopod indistinctly 3-segmented and distal 2 segments partly fused, proximal segment with seta on middle of inner margin, middle segment with 3 inner setae, distal segment with 4 distal setae.

***Mandible*** (Fig. 2B, C) gnathobase with 4 well-chitinized teeth, dorsalmost bifurcate; row of setules on dorsal margin of gnathobase; basis unarmed; endopod rudimentary, unsegmented, with 2 setae; exopod 5-segmented, setal formula 1, 1, 1, 1, 1.

***Maxillule*** (Fig. 2D) with 5 long spines, 1 short spine and row of long setules on praecoxal arthrite; coxal endite with 1 seta; coxal epipodite with 6 setae; proximal and distal basal endites without setae; endopod unsegmented with 2 setae; exopod with 3 setae.

***Maxilla*** (Fig. 2E) with praecoxal and coxal endites having 2, 2, 2 and 2 setae, respectively; basis having 1 heavily-chitinized spine with row of long spinules midway; endopod 4-segmented, setal formula 1, 3, 2, 2 [Maxilla with praecoxal and coxal endites having 2, 2 setae, respectively; basal endites having 2, 2 setae, respectively; endopod 5-segmented, setal formula 1, 1, 3, 2, 2; first endopodal segment having 1 heavily-chitinized spine with row of long spinules midway (the homology by Ferrari and Ivanenko 2001, 2008)].

***Maxilliped*** (Fig. 2F) syncoxal endites with 0, 1, 0, and 2 setae, respectively; rows of long setules on inner margin of syncoxa and basis; basis with 2 setae midway; first endopodal segment partly fused to basis; first to sixth endopodal segments with 1, 4, 3, 2, 2, and 4 setae, respectively [Maxilliped praecoxal endites with 0, 1, and 0 setae, respectively; coxal endite with 1 seta; rows of long setules on inner margin of coxa and basis; basis with 2 setae midway, and 1 seta distally; first to fifth endopodal segments with 4, 3, 2, 2, and 4 setae, respectively (the homology by Ferrari and Ivanenko 2001, 2008)].

**Figure 3.**
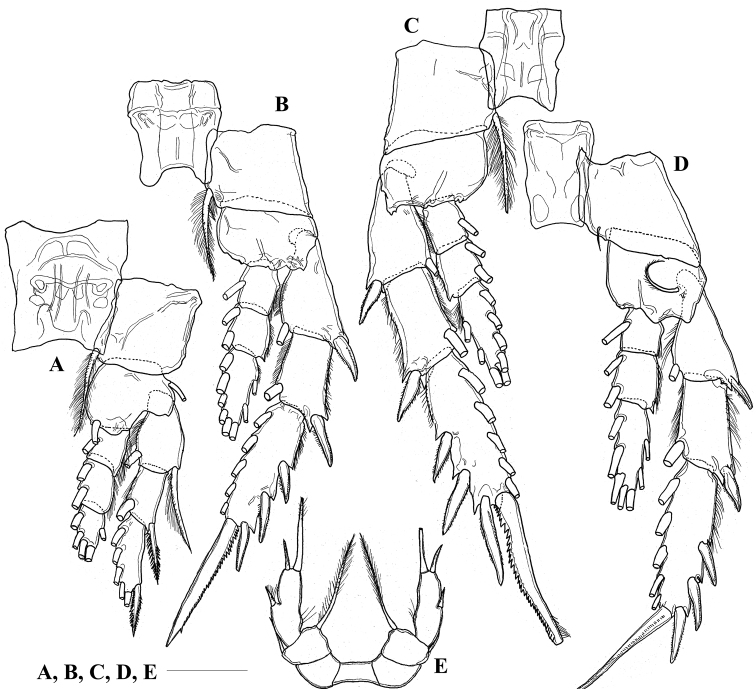
*Pilarella compacta* sp. nov., adult female, paratype **A** leg 1, posterior side **B** leg 2, posterior side **C** leg 3, posterior side **D** leg 4, posterior side **E** fifth legs, posterior side. Scale bar: 0.1 mm.

Seta and spine formula of ***legs 1–4*** as shown in Table 2. Leg 1 (Fig. 3A) with medial setules on proximal 2 segments of exopod; proximal 2 segments of endopod with lateral setules. Legs 2 and 3 (Fig. 3B, C) with medial setules on proximal 2 segments of exopod; distal 2 segments of exopod and all segments of endopod with lateral setules. Leg 4 (Fig. 3D) with medial setules on proximal 2 segments of exopod; distal 2 segments of exopod and proximal 2 segments of endopod with lateral setules.

***Leg 5*** (Fig. 3E) uniramous, 3-segmented; basis broad, approximately 0.6 times as long as wide, with long plumose seta medially and short seta laterally; exopod unsegmented, approximately 3 times longer than wide, with 3 medial setules, 1 lateral spine and 2 terminal spines.

**Figure 4.**
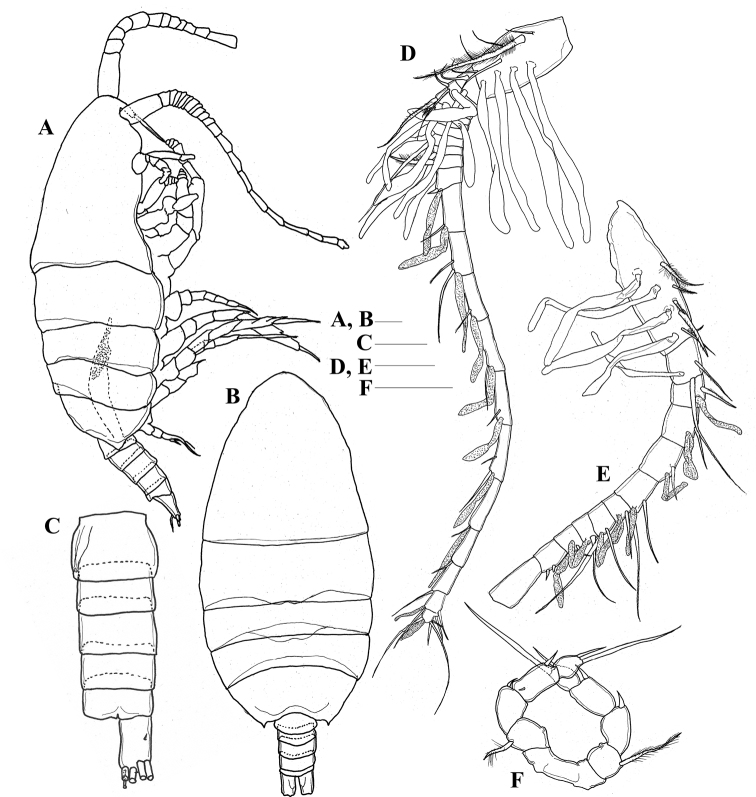
*Pilarella compacta* sp. nov., adult male, allotype **A** lateral habitus **B** dorsal habitus **C** urosome, ventral view **D** right antennule **E** left antennule **F** Fifth legs, anterior view. Scale bars: 0.1 mm.

**Adult male. *Body*** (Fig. 4A, B) compact; cephalosome separated from first pediger; fourth and fifth pedigers completely fused; posterolateral corners of prosome with small triangular process extending posteriorly. Rostrum similar to that of female. Urosome (Fig. 4C) 5-segmented; gonopore located on right side of genital somite; caudal rami similar to female, except for lacking inner setules.

***Antennules*** (Fig. 4D, E) asymmetrical. Right antennule (Fig. 4D) 21-segmented, ancestral segments XXIV and XXV partly fused, exceeding middle of first pediger, fusion pattern and armature of segments as follows: I-IV–6+4ae, V–1+1ae, VI–2+1ae, VII–1+1ae, VIII–1+1ae, IX–1+1ae, X–1ae, XI–1+1ae, XII–1+1ae, XIII–1+1ae, XIV–1+1ae, XV–1+1ae, XVI–2+1ae, XVII–1+1ae, XVIII–1+1ae, XIX–1+1ae, XX–2+1ae, XXI–1+1ae, XXII–1, XXIII–1, XXIV-XXVIII–7+2ae. Left antennule (Fig. 4E) damaged; ancestral segments XV–XXVIII not observed, fusion pattern and armature of I–XV as follows: I-IV–6+4ae, V–2+1ae, VI–1+1ae, VII–1+1ae, VIII–1+1ae, IX–1+1ae, X–2+1ae, XI–2+1ae, XII–1+1ae, XIII–1+1ae, XIV–2.

***Antenna, mandible, maxillule, maxilla, maxilliped and legs 1–4*** similar to those of female.

***Leg 5*** (Fig. 4F) uniramous, 5-segmented; basis with plumose seta laterally; exopod 3-segmented, proximal 2 segments with lateral spine, distal segment with 2 terminal setae.

###### Etymology.

The specific name of the new species is derived from the Latin adjective *compactus* meaning “stocky” to denote the habitus of the present new species.

###### Remarks.

The present new species falls within the diagnosis of the monotypic *Pilarella* (Alvarez 1985) except for the following features: (1) left antennule not reaching the caudal rami; (2) short caudal rami; (3) 6 spines on the maxillular praecoxal arthrite (5 in the previous diagnosis); and (4) 6 setae on the maxillular coxal epipodite (5 in the previous diagnosis). For the differences from other genera, see generic remarks in the present study.

*Pilarella compacta* sp. nov. differs from *P. longicornis* Alvarez, 1985 as follows: (1) the body is more compact (vs. more slender in *P. longicornis*); (2) the left antennule of the female is short, and reaches the posterior border of genital double-somite (vs. reaching the posterior margin of caudal rami in *P. longicornis*); (3) the caudal rami are short, approximately 1.5 times longer than wide (vs. long, 4.3 longer than wide in *P. longicornis*), (4) the mandibular endopod has 2 setae (vs. 1 in *P. longicornis*), (5) the coxal epipodite of maxillule has 6 setae (vs. 5 in *P. longicornis*), (6) there is a basal lateral seta on leg 4 (vs. seta absent in *P. longicornis*).

###### Phylogenetic analysis.

**Figure 5.**
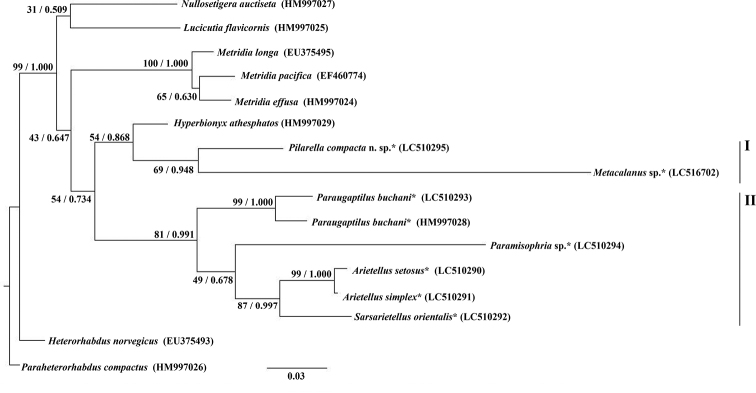
Maximum Likelihood tree based on partial 28S nuclear ribosomal DNA sequences of the superfamily Arietelloidea. Left and right Arabic numerals on nodes indicate bootstrap values and posterior probabilities, respectively. Scale bar shows nucleotide changes per site. Asterisks indicate arietellid species.

The Maximum Likelihood tree based on 16 species of the superfamily Arietelloidea is shown in Figure 5. Both ML and BI trees showed two clades in Arietellidae. In clade I, *Pilarella compacta* sp. nov. and *Metacalanus* sp. were placed in the same clade (BP = 69%; PP = 0.948). *Hyperbionyx athesphatos* (HM997029) (belonging to the Hyperbionychidae) and clade I formed a cluster with a low bootstrap value (BP = 54%; PP = 0.868). In clade II, four arietellid genera (*Paraugaptilus*, *Paramisophria*, *Sarsarietellus*, and *Arietellus*) were grouped in the same cluster with high bootstrap value (ML = 81%; PP = 0.991), and *Arietellus* was placed in the same clade as *Sarsarietellus* with a high bootstrap value (BP = 87%; PP = 0.997). The sequence of *Paraugaptilus buchani* (LC510293) in this study showed a lower identity of 96% to *P. buchani* (HM997028) in the DDBJ/EMBL/GenBank databases, which differed by five gaps and 19 single nucleotide polymorphisms (SNP).

### Key to genera of the family Arietellidae

The homology of the maxilla and maxilliped by Ferrari and Ivanenko (2001, 2008) is shown in parenthesis.

**Table:** 

1	Maxillule located anterior to maxilla; maxillular coxal epipodite with 5–9 setae	**2**
–	Maxillule located posterior to maxilla and maxilliped; maxillular coxal epipodite without setae	***Griceus* Ferrari & Markhaseva, 2000**
2	Third exopodal segment of leg 1 with 1 outer spine	**3**
–	Third exopodal segment of leg 1 with 2 outer spines	**6**
3	Maxillular praecoxal arthrite with 5–6 elements	**4**
–	Maxillular praecoxal arthrite with 0–2 elements	***Metacalanus* Cleve, 1901**
4	Distal endite on maxillary praecoxa with 2 setae (coxal endite on maxilla with 2 setae)	**5**
–	Distal endite on maxillary praecoxa with 1 seta (coxal endite on maxilla with 1 seta)	***Scutogerulus* Bradford, 1969**
5	Third ancestral segment on antennary exopod without seta; legs 1–4 symmetrical; female leg 5 with 1 basal medial seta, 1 exopodal lateral spine and 2 exopodal distal spines	***Pilarella* Alvarez, 1985**
–	Third ancestral segment on antennary exopod with seta; legs 1–4 asymmetrical; female leg 5 with no basal medial seta, 2 exopodal lateral spines and 2 exopodal distal spines	***Metacalanalis* Ohtsuka, Nishida & Machida, 2005**
6	Maxillular endopod absent or unsegmented with 0–3 setae	**7**
–	Maxillular endopod 3-segmented with 6 setae	***Rhapidophorus* Edwards, 1891**
7	Maxillule with 6 setae on coxal epipodite	**8**
–	Maxillule with 8–9 setae on coxal epipodite	**9**
8	Antennary exopod indistinctly 10-segmented; maxillule with strongly serrate spines on coxal arthrite; innermost seta on fifth endopodal segment of maxilliped long; outermost seta on sixth endopodal segment of maxilliped not reduced (innermost seta on fourth endopodal segment of maxilliped long; outermost seta on fifth endopodal segment of maxilliped not reduced)	***Crassarietellus* Ohtsuka, Boxshall & Roe, 1994**
–	Antennary exopod indistinctly 8-segmented; maxillule with weakly serrate spines on coxal arthrite; innermost seta on fifth endopodal segment of maxilliped short; outermost seta on sixth endopod segment of maxilliped reduced (innermost seta on fourth endopodal segment of maxilliped short; outermost seta on fifth endopod segment of maxilliped reduced)	***Campaneria* Ohtsuka, Boxshall & Roe, 1994**
9	Innermost seta on fourth and fifth endopodal segments of maxilliped ordinary (Innermost seta on third and fourth endopodal segments of maxilliped ordinary)	**10**
–	Innermost seta on fourth and fifth endopodal segments of maxilliped vestigial (Innermost seta on third and fourth endopodal segments of maxilliped vestigial)	***Arietellus* Giesbrecht, 1892**
10	Ancestral segment X on antennary exopod with 3 elements	**11**
–	Ancestral segment X on antennary exopod unarmed	***Paraugaptilus* Wolfenden, 1904**
11	Leg 4 without inner coxal seta; second segment of antennary endopod with 3 inner setae midway	**12**
–	Leg 4 with inner coxal seta; second segment of antennary endopod with 2 inner setae midway	***Paraugaptiloides* Ohtsuka, Boxshall & Roe, 1994**
12	Antennulary segments XXV and XXVI fused; maxillary basal spine without spinules (spine on first endopodal segment of maxilla without spinules); basal inner process on female leg 5 with 0–2 setae	**13**
–	Antennulary segments XXV and XXVI separated; maxillary basal spine with spinules (spine on first endopodal segment of maxilla with spinules); basal inner process on female leg 5 with 4 setae	***Sarsarietellus* Campaner, 1984**
13	Female genital double-somite with single copulatory pore; third ancestral segment on exopod of female leg 5 with at most 3 elements and 1 process	***Paramisophria* T. Scott, 1897**
–	Female genital double-somite with paired copulatory pores; third ancestral segment on exopod of female leg 5 with 1 outer, 1 subterminal and 2 terminal elements; male unknown	***Protoparamisophria* Ohtsuka, Nishida & Machida, 2005**

### Key to species of *Pilarella*

**Table:** 

1	Caudal rami approximately 1.5 times longer than wide; mandibular endopod with 2 setae; maxillular coxal epipodite with 6 setae; female left antennule reaching posterior border of genital double-somite	***P. compacta* sp. nov.**
–	Caudal rami approximately 4 times longer than wide; mandibular endopod with 1 seta; maxillular coxal epipodite with 5 setae; female left antennule reaching posterior edge of caudal rami	***P. longicornis* Alvarez, 1985**

## Discussion

This study is the first report of a male *Pilarella*. The male antennule of *Pilarella compacta* sp. nov. has long, tape-like aesthetascs on the basal part. Similar antennules were found in a male *Metacalanus acutioperculum* and *Metacalanus adriaticus* (Ohtsuka 1984; Kršinić and Boxshall 2021). The segmentation and setation of the male antennule are similar to those of *Crassarietellus* and more segments are retained than in the diagnosis of *Metacalanus* (Ohtsuka et al. 1994). The male leg 5 has the same segmentation as in *Metacalanus aurivilli* (Chen and Zhang 1965), and corresponds to the *Metacalanus*-clade in the reduction of endopod (Ohtsuka et al. 1994). These features of the male *Pilarella* suggest its close relation to *Crassarietellus* and *Metacalanus*, which belong to the *Metacalanus*-clade in Ohtsuka et al. (1994).

As regards the molecular analysis, clades I (*Metacalanus*, *Paramisophria*, and *Pilarella*) and II (*Arietellus*, *Paraugaptilus*, and *Sarsarietellus*) in Figure 5 correspond to two lineages, the *Metacalanus*- and *Arietellus*-clades, which were discussed by Ohtsuka et al. (1994, 2005) and Soh et al. (2013). Although Ohtsuka et al. (1994, 2005) considered *Paramisophria* to be in the *Metacalanus*-clade, Soh et al. (2013) regarded the position of *Paramisophria* as equivocal based on their cladistic analysis. In the present study, *Paramisophria* was included in clade II (*Arietellus*-clade), although with low bootstrap value and posterior probability (BP = 49%, PP = 0.678).

*Hyperbionix*, belonging to the Hyperbionychidae, was clustered in clade I and included in the lineage of the Arietellidae (Fig. 5). However, estimating the phylogenetic position of the Hyperbionychidae is difficult because the cluster of Hyperbionychidae + clade I was supported by a low bootstrap value (BP = 54%). Blanco-Bercial et al. (2011) indicated the close relationship between *Paraugaptilus buchani* (Arietellidae) and *Hyperbionyx athesphatos* (Hyperbionychidae) in their molecular phylogeny. Ohtsuka et al. (1993) described many autapomorphic characteristics in the Hyperbionychidae, and Soh (1998) indicated that the Hyperbionychidae has some plesiomorphic characteristics that are not observed in the Arietellidae. These studies suggested that the Hyperbionychidae is closely related to the Arietellidae but is not derived from the Arietellidae.

In clade II (*Arietellus*-clade), a comparison of the *28S* sequences of *Paraugaptilus buchani* between the present (material from the western North Pacific) and a previous study (material from the western North Atlantic; Blanco-Bercial et al. 2011) showed a low concordance rate (96%), differing by five gaps and 19 SNPs. However, no differences in morphological characteristics were observed between the *Paraugaptilus buchani* material collected from the western North Pacific in the present study and the original description of the species based on Atlantic material (Deevey 1973). As a possible explanation of this discordance, we consider it as due to intra-specific variation. Differences in genetic structure among populations of the same species have been reported in many other calanoids (Bucklin et al. 2003). In our case, it may be caused by the significant geographic distance separating the Pacific and the Atlantic locations where the material was collected.

The present study suggests a genetically closer relationship of *Arietellus* to *Sarsarietellus* based on high bootstrap value and posterior probability (BP = 87%, PP = 0.997) rather than to *Paraugaptilus*, as previous studies based on morphological features pointed out (Ohtsuka et al. 1994, 2005; Soh et al. 2013). Furthermore, *Arietellus* and *Sarsarietellus* also share a synapomorphic fused right and left copulatory pores (Ohtsuka et al. 1994; Ohtsuka et al. 2004). These two closely related genera, *Arietellus* and *Sarsarietellus*, are distributed across the pelagic realm and the deep-sea hyperbenthic layers, respectively (Ohtsuka et al. 1994). Ohtsuka et al. (2005) suggested that, based on morphology, the two pelagic genera (*Arietellus* and *Paraugaptilus*) could have diverged from a hyperbenthic ancestor. However, the present study shows that *Arietellus* (pelagic genus) is closer to *Sarsarietellus* (hyperbenthic genus) rather than to *Paraugaptilus*, and that *Arietellus* and *Paraugaptilus* could have colonized the pelagic realm independently, or that *Sarsarietellus* secondarily turned back to the deep-sea hyperbenthic layers. In conclusion, the molecular phylogenetic relationships of the Arietellidae supported the *Metacalanus*- and *Arietellus*-clades as depicted by Ohtsuka et al. (1994, 2005) and Soh et al. (2013), and provide new information about other possible colonization routes followed by members of the *Arietellus*-clade.

## Supplementary Material

XML Treatment for Pilarella

XML Treatment for Pilarella compacta
